# Identifying ELIXIR Core Data Resources

**DOI:** 10.12688/f1000research.9656.2

**Published:** 2017-03-09

**Authors:** Christine Durinx, Jo McEntyre, Ron Appel, Rolf Apweiler, Mary Barlow, Niklas Blomberg, Chuck Cook, Elisabeth Gasteiger, Jee-Hyub Kim, Rodrigo Lopez, Nicole Redaschi, Heinz Stockinger, Daniel Teixeira, Alfonso Valencia

**Affiliations:** 1 SIB Swiss Institute of Bioinformatics, Lausanne, Switzerland; 2 European Molecular Biology Laboratory, European Bioinformatics Institute (EMBL-EBI), Cambridge, UK; 3 ELIXIR, Cambridge, UK; 4 SIB Swiss Institute of Bioinformatics, Geneva, Switzerland; 5 Centro Nacional de Investigaciones Oncológicas, Madrid, Spain

**Keywords:** ELIXIR, Sustainability, Data resources, Indicators, Capacity building, Infrastructure, Bioinformatics, Life sciences

## Abstract

The core mission of ELIXIR is to build a stable and sustainable infrastructure for biological information across Europe. At the heart of this are the data resources, tools and services that ELIXIR offers to the life-sciences community, providing stable and sustainable access to biological data. ELIXIR aims to ensure that these resources are available long-term and that the life-cycles of these resources are managed such that they support the scientific needs of the life-sciences, including biological research.

ELIXIR Core Data Resources are defined as a set of European data resources that are of fundamental importance to the wider life-science community and the long-term preservation of biological data. They are complete collections of generic value to life-science, are considered an authority in their field with respect to one or more characteristics, and show high levels of scientific quality and service. Thus, ELIXIR Core Data Resources are of wide applicability and usage.

This paper describes the structures, governance and processes that support the identification and evaluation of ELIXIR Core Data Resources. It identifies key indicators which reflect the essence of the definition of an ELIXIR Core Data Resource and support the promotion of excellence in resource development and operation. It describes the specific indicators in more detail and explains their application within ELIXIR’s sustainability strategy and science policy actions, and in capacity building, life-cycle management and technical actions. The identification process is currently being implemented and tested for the first time. The findings and outcome will be evaluated by the ELIXIR Scientific Advisory Board in March 2017.

Establishing the portfolio of ELIXIR Core Data Resources and ELIXIR Services is a key priority for ELIXIR and publicly marks the transition towards a cohesive infrastructure.

## Introduction

ELIXIR is an intergovernmental organisation which builds on existing data resources and services within Europe. It follows a Hub and Nodes model, with a single Hub located in Hinxton, United Kingdom, and a growing number of Nodes located at centres of excellence throughout Europe. Governments and ministries of countries are members of the ELIXIR consortium, and the scientific community in each member country develops their national Node.

The core mission of ELIXIR is to build a stable and sustainable infrastructure for biological information across Europe. At its heart are the data resources, tools and services that ELIXIR Nodes offer to the life-science community, providing stable and sustainable access to biological data.

ELIXIR resources vary from archives, or deposition databases, which contain research data outputs such as DNA sequences, to highly dynamic knowledge bases which aggregate, process and visualize research data, often adding layers of value through manual curation by highly qualified personnel. ELIXIR aims to ensure that these resources are available long-term and that their life-cycles are managed so that they support the scientific needs of life-sciences and biological research.

Over 500 data resources exist in Europe ^1^. Only a small fraction of these have institutional support and long-term funding commitments. The fact that the mid- and long-term survival of many crucial bioinformatics resources is not guaranteed threatens the foundations of academic and industrial life-science activities, and risks the loss of an immense wealth of biological and medical information, and the associated investments.

**Figure 1.**
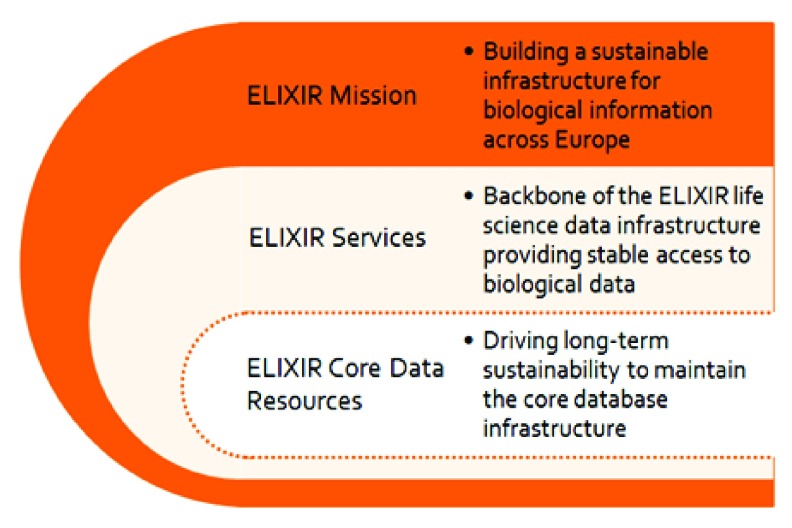
The place of ELIXIR Services and ELIXIR Core Data Resources within ELIXIR’s mission.

Identifying ways to assess the quality and impact of these crucial data resources will (a) promote excellence in resource development and operation to support capacity building through spreading best practice, and (b) provide a basis for technical and science policy actions required to support the long-term sustainability of the resources that form the backbone of bioinformatics infrastructure ( Figure 1).

**Figure 2.**
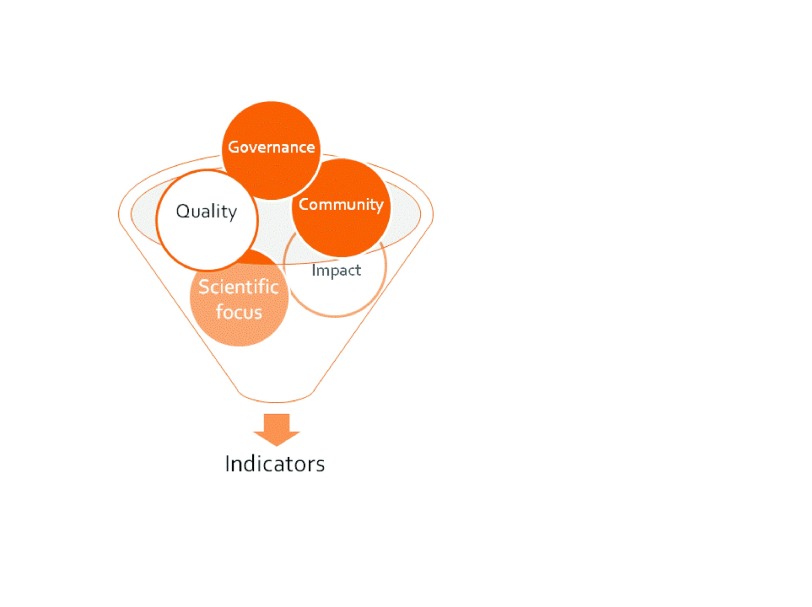
A carefully chosen basket of qualitative and quantitative indicators for bioinformatics resources.

**Figure 3.**
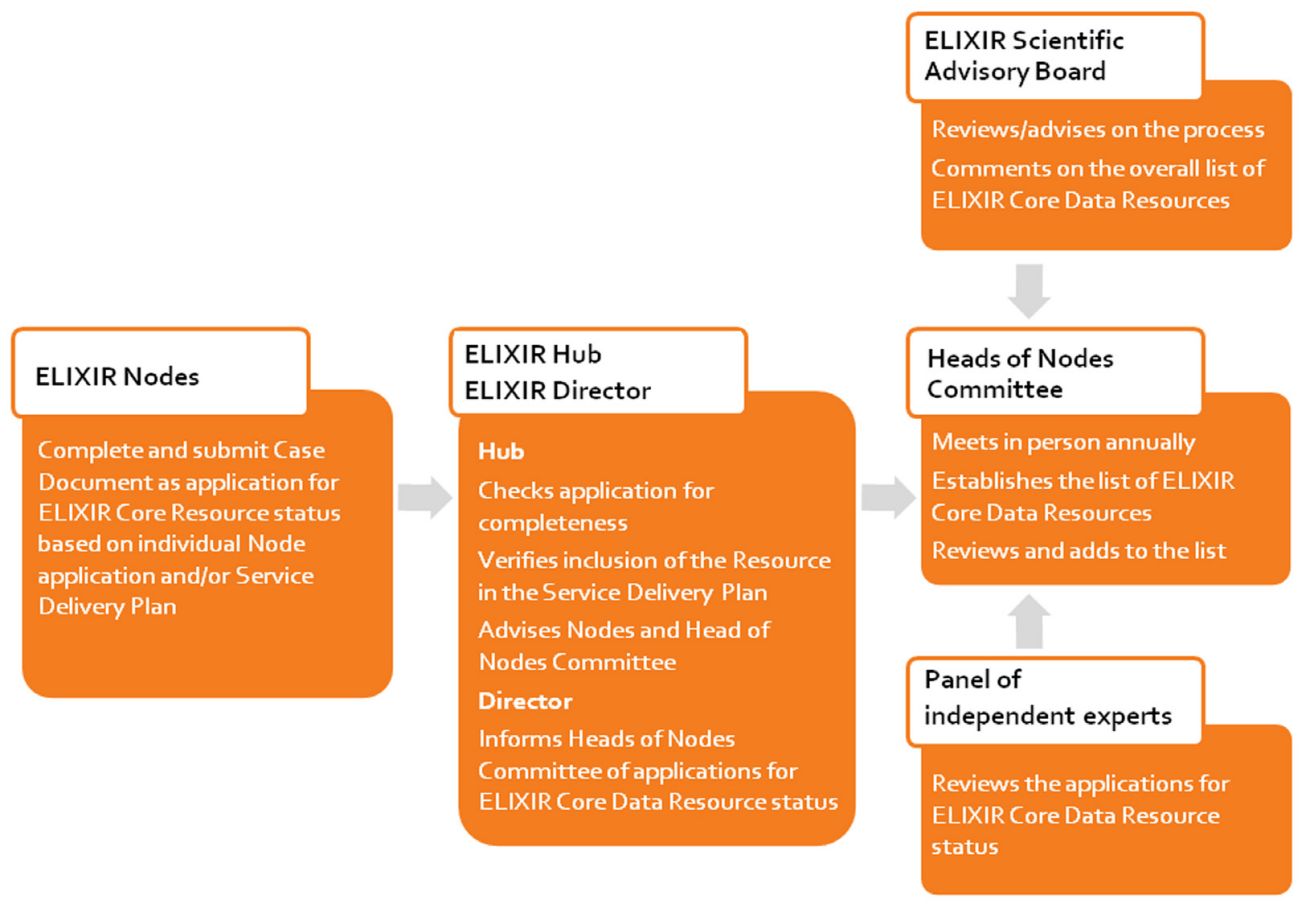
Process for identifying ELIXIR Core Data Resources.

The proposal for establishing ELIXIR Services and ELIXIR Core Data Resources was put to the ELIXIR Scientific Advisory Board (SAB) in December 2014 ^2^. This paper describes how to put the proposal into practice and provides guidelines for the implementation of life-cycle management.

ELIXIR Nodes define, through their Node applications and Service Delivery Plans or Work Programme, a set of services and data resources that are offered to the research community, the ELIXIR Services. These resources form the backbone of the life-science data infrastructure.

ELIXIR Core Data Resources are defined as a set of European data resources that are of fundamental importance to the wider life-science community and the long-term preservation of biological data. They provide complete collections of generic value to life-science, are considered an authority in their field with respect to one or more characteristics, and show high levels of scientific quality and service. Thus, ELIXIR Core Data Resources are of wide applicability and usage.

ELIXIR Core Data Resources tend to be well-known within the life-science community and are known to key stakeholders such as funders and journals. ELIXIR Core Data Resources are well maintained with a professional service delivery plan based on well-established life-cycle management processes and well-understood dependencies with related data resources. The ELIXIR Core Data Resources coexist with a broader range of databases with diverse motivations, often specialising in a particular scientific topic.

The ELIXIR Core Data Resources will form the focal point of technical and science policy actions to drive long-term sustainability. Transparent indicators for the ELIXIR Core Data Resources will also provide strategic intelligence on resource quality and impact, notably to policy makers and funders.

Through the ELIXIR Scientific Programme and ELIXIR-EXCELERATE grant, the infrastructure will deliver and enable a range of initiatives to support and strengthen the ELIXIR Services and ELIXIR Core Data Resources. ELIXIR Services and ELIXIR Core Data Resources will be the most widely used and outwardly visible part of ELIXIR. Establishing the portfolio of these data resources and services is the key priority for ELIXIR and publicly marks the transition towards a cohesive infrastructure. The identification process is currently being implemented and tested for the first time. The findings and outcome will be evaluated by the ELIXIR Scientific Advisory Board in March 2017. Through the establishment of the ELIXIR Services portfolio, ELIXIR also aims to support and implement best practice in resource management and bring European bioinformatics resources to the next level, building confidence among users.

## Methods

### Measuring the quality and impact of data resources: key indicators

In their report on the role of metrics in research assessment and management in the United Kingdom ^3^, Wilsdon *et al.* highlight that the term ‘metric’ may be misunderstood. For example, the number of citations received by a publication is a citation metric, as it does not directly measure the impact of that researcher’s work.

They therefore suggest that the term ‘indicator’ is used in contexts in which there is the potential for confusion ( Table 1). An ‘indicator’ is defined as a measurable quantity that substitutes for something less readily measurable and is presumed to associate with it without directly measuring it. Citation counts could be used as indicators for the scientific impact of journal articles, even though scientific impacts can occur in ways that do not generate citations. We therefore use the term ‘indicators’ throughout.

### Life-cycle management of ELIXIR Services

This section outlines the framework and stages for life-cycle management of the ELIXIR Services ( Table 1). This framework will be implemented through the ELIXIR-EXCELERATE Node Capacity Building and Communities of Practice and Training Programme work packages, strengthening the ELIXIR infrastructure by creating a pathway to excellence.

**Table 1.** Stages of the technical life-cycle of ELIXIR Services.

Stage	Definition	Status
**Emerging**	A resource in active development towards maturity. Emerging Services may have lower reliability compared to Mature Services and go through more changes in their presentation and APIs. ‘Emerging’ status should not exceed 2 years. If an Emerging Service does not become Mature, an “end of service” date should be prominently displayed for at least 6 months before it is withdrawn.	ELIXIR Emerging Services
**Mature**	An ELIXIR Service that has passed the development stage. It is reliable and active, i.e. new data are being added. If feasible, major changes to its API and/or user interface that may break existing functionality and/or are not fully back-compatible, are notified at least 6 months in advance. A Mature Service relies only on other Mature or Legacy Services. Exceptionally, a Mature Service may rely on an Emerging Service that is close to becoming mature. Service withdrawal should be notified at least 1 year in advance, during which time the Service has Legacy status.	ELIXIR Services
**Legacy**	A previously Mature Service scheduled for archiving or decommissioning. A Service must spend at least 1 year in the Legacy state before final withdrawal. Reliability should be at the same level as Mature Services, but compromises on content (e.g. data not updated, no new content is added) are allowed.	ELIXIR Services – Legacy

The agreed set of indicators for the ELIXIR Core Data Resources sets quality standards that guide and inform the managers of Emerging Services in the development of their Resource towards an ‘ELIXIR Service’ status.

Monitoring of usage trends and the scientific impact of the ELIXIR Services provides information to support their management, contributing to the maintenance of the ELIXIR Service status, or – where appropriate – leading a resource towards the Legacy stage ( Table 1).

### Five categories of indicators, reflecting the multiple facets of data resources

Identification of the ELIXIR Core Data Resources involves a careful evaluation of the multiple facets of the data resources.

Indicators are grouped in five categories:

(1) Scientific focus and quality of science
(2) Community served by the resource
(3) Quality of service
(4) Legal and funding infrastructure, and governance
(5) Impact and translational stories

When collecting and interpreting indicators, it is important to articulate the methods used and, where possible, standardise terminology. This facilitates the understanding of the indicators and avoids misinterpretation across different Nodes.

**(1) Scientific focus and quality of science**

This includes the inherent scientific quality of the data and of the metadata, and its uniqueness and comprehensiveness. Also included are benchmarking against other resources, and whether the resource is an authority in its field.

A differentiation should be made between archival or deposition databases that receive and archive *de novo* data sets and well-structured metadata deposited by scientists, and added-value databases or knowledge bases, which are based on the archival data and add substantial value through expert curation, annotation of metadata, sophisticated data processing and/or data integration. The curation effort and outputs linked to a resource are an important measure of its quality.

**(2) Community**

This category reflects the size and the measured demand of the communities that are served by the resource: web statistics, user reach, and international use. The community that is served can be the depositors, as some resources are vital for deposition and/or the end-users. The community can be identified and measured in different ways, such as access to URLs, to download servers, and through APIs, and also through the citation of data and data resources in publications.

In addition, certain resources play a foundational role to derived services and data-driven research. Their data are distributed to many other resources and/or services that rely on their existence.

The scientific context in which the resource operates should be taken into account. A resource that serves a small scientific community may not have as many users as a resource serving a broader interest, and yet it may reach 90% of the community it supports (coverage) and be crucial for the scientific work of that community.

**(3) Quality of service**

Certain service levels and reliability can be quantified with specific technical indicators such as: the uptime of the resource; response times; availability and periodic application of meaningful and automated tests; user support and related training; use of community-recognised standards; diversity of data retrieval mechanisms; and other services. Usually, this requires a quality-assurance process during service development and operation. The Accelerating the ELIXIR Training Programme and the ELIXIR Training Platform will support resources delivering training, as well as provide good-practice guidelines and systems for evaluation.

**(4) Legal and funding infrastructure, and governance**

As stable research infrastructures, Core Data Resources can demonstrate that they have a sound legal, funding and governance structure.

A viable resource has a suitable legal framework (clear terms of use, licensing, data security, ethical compliance, etc.). Open data is a critical driver for life-sciences research and therefore for ELIXIR, but the policy for data access must be considered in view of resource funding. Longevity can be gauged through institutional support, funding schemes and the duration of financial stability. Core Data Resources will have demonstrated transition through different funding sources. A strong governance structure includes an international, independent Scientific Advisory Board (SAB), which allows community input and provides permanent oversight.

**(5) Impact and translational stories**

Impact evaluation attempts to provide a definitive answer to the question of whether the resource is meeting its objective of fulfilling a specific need of the scientific community. The translational stories relate to the role of the resource in accelerating science and are thus a very important indicator.

Impact evaluation attempts to assess whether the Resource is meeting its objective of fulfilling a specific need. In the UK, the HM Treasury’s Magenta Book ^4^ provides guidelines for policy makers and analysts on how policies and projects should be assessed and reviewed. According to this guidance, the key characteristic of a good impact evaluation is that it recognises that most needs can be met by a range of elements, not just the project in question. To test the extent to which the Resource is responsible for meeting the need, it is necessary to estimate – usually on the basis of a statistical analysis of quantitative data – what would have happened if the Resource had not existed. This is known as the counterfactual. Establishing the counterfactual is not easy, since by definition it cannot be observed. A strong evaluation is successful in isolating the effect of the Resource from all other potential influences, thereby producing a good estimate of the counterfactual.

When communicating the impact of ELIXIR’s resources and their role in accelerating science to funders and the public, the indicators should be relevant to the audience. This can be done by presenting them within a context that is readily understandable.

### Detailed description of the indicators and related methodology

One of the challenges of data-intensive science is to facilitate knowledge discovery by assisting humans and machines in their discovery of, and access to, scientific data. FAIR is a set of guiding principles to make data Findable, Accessible, Interoperable, and Reusable ^6^. These indicators will be used to demonstrate that ELIXIR Core Data Resources are compatible with the FAIR data principles. The Table in Box 1 maps indicators to corresponding FAIR criteria.

Box 1 describes the indicators used in each of the categories above.

Box 1. **Quantitative and qualitative indicators for ELIXIR Core Data Resources**

ELIXIR Core Data Resources are defined as a set of European data resources that are of fundamental importance to the broad life-science community and the long-term preservation of biological data.

A set of key indicators may be used to make a case for a Core Data Resource. Indicators aim to reflect the essence of the definition of an ELIXIR Core Data Resource and support the promotion of excellence in resource development and operation.

Indicators are grouped in five categories:

(1) **Scientific** focus and quality of science
(2) **Community** served by the resource
(3) **Quality** of service
(4) **Legal** and funding infrastructure, and governance
(5) **Impact** and translational stories.

The indicators recognise the heterogeneous nature of biological data, and the diversity of the supporting data resources, use cases, and communities served. Indicators can be used to measure technical and/or scientific readiness of a resource compared to defined quality standards.

The Table below maps indicators to corresponding FAIR criteria.

As the context of a core resource is critical to understanding its importance, indicators alone are not sufficient. Qualitative evidence is needed so that the resource can be reviewed throughout its life-cycle through the expert judgment of the ELIXIR Heads of Nodes and Scientific Advisory Boards.

**Indicators and Related Information.**

All elements in sections 1–4 require a response.

**Quantitative indicators are underlined.**

**1. Scientific focus and quality**

a. *Archives vs knowledge bases*: is the resource archival (taking submissions) or a knowledge base (added-value)?
b. *Scope statement*: describe the scientific coverage and comprehensiveness of the resource. For example, all species or a subset of species, families, outputs from a particular experimental method? What is position of the resource with respect to other similar data resources?
c. *International dimension*: does the resource have a global footprint? (Demonstrated through, for example, an international consortium delivering the resource, geographical diversity in sources of submissions, global literature curated, international diversity of delivery partners and/or funders)
d. *Staff effort:* number of FTEs per year for the past 2–3 years
  i. Curators
    ❑ support for submission adherence to metadata requirements? (see also 3d)
    ❑ support for extraction of information from the scientific literature?
  ii. Bioinformaticians
  iii. Technical staff

**2. Community**

a. *Overall usage:* what is the usage of the resource for the past 2–3 years?
i. Access via a web browser: number of visits, unique visitors, hits, and page views ^1^
ii. Access via additional access methods: visits, unique visitors, hits, and downloads (includes FTP downloads and programmatic access)

b. *Potential usage:* what is the estimated size of the global potential user community?

c. *Usage in research as measured through citation in the literature:*
  i. Citation of a resource name: the number of times the resource name is mentioned in scientific articles per year (in Europe PMC)
  ii. Citation of data of a resource: the number of times accession numbers from the resource are mentioned or cited in research articles (in Europe PMC)
  iii. Key publications describing the resource list (e.g. publications in NAR Database issue) and the number of citations (in Europe PMC).

d. *Dependency of other resources:* do other resources have a dependency on the resource described here to provide that service (i.e. what is the reach-through)?

**3. Quality of service**

a. *Identifier use*: does the resource provide persistent and unique identifiers?
b. *Data throughput:* number of entries, depositions (records or bytes ingested per year), records processed, genomes assembled, etc. per year, for past 2–3 years.
c. *Technical performance*:
  i. Uptime: percentage availability per month for a sample of key web pages (or similar) over the past 12 months (e.g. search results, homepage, data record pages).
  ii. Response times of key web pages.
d. *Use of standards*: which community-recognised standards are used for metadata and data (e.g. MIAME, JATS, INSDC features, ontologies)? Provide a link to documentation.
e. *Links to documentation of provenance*: does the resource link to the scientific literature for provenance of facts or biological context?
f. *Data availability* - access services and formats
  i. Data sharing services: list services through which data is shared (e.g. website, APIs, FTP, TripleStore)
  ii. Data sharing formats: list formats for available data (e.g. plain text, FASTA, XML, RDF, Dublin Core, tsv, JSON)
g. *Customer service*
  i. Helpdesk: does the resource run a helpdesk?
  ii. User feedback: does the resource seek and incorporate user input into service design decisions?
  iii. Training: does the resource undertake training?

**4. Legal and funding infrastructure, and governance**

a. *Scientific Advisory Board:* does the resource have an international, independent Scientific Advisory Board
b. *Open Science:* does the resource have a legal framework that supports Open Science? e.g. open licenses or a public statement of open terms of use.
c. *Privacy policy:* does the resource have a publically available privacy policy in which security around personal data and cookies are described?
d. *Ethics policy*: does the resource have an ethics policy that complies with all relevant international standards and best practices?
e. *Sustainable support and funding:* demonstrate the past and future funding and/or other commitments that support the resource by the host institution and/or other entities.

**5. Impact and translational stories**

a. *Counterfactual*: what would be the impact on the scientific community if the resource had not existed, or were to disappear and not be replaced? Is the resource globally unique? What would the impact on other dependent resources be?
b. *Accelerating science*: how does the resource accelerate science? For example, does the resource set standards; promote reuse of data or software; promote research efficiencies; extend technical products in other areas?
c. *Translational data*: are there ‘translational’ figures familiar to the audience that will help them grasp the core nature of the resource?

**Definition of terms used to measure overall resource usage (see 2.a)**

**Visits:** a visit, or session, is a set of requests/interactions by a uniquely identified client within a specific time (typically, 30 minutes). The number of visits/sessions is a measure of website traffic.

**Unique Visitors:** the number of visitors (unique IP addresses, unique visitors, or visitors) measures how many individuals access a website in a specified time, regardless of how often they visit. It can be determined in different ways. For example, number of: unique IP addresses, user cookies, unique IP addresses + user agent (a ‘user agent’ is the client that is used to access a web site.

**Hits:** can be used to analyse usage trends of a web resource. Hits measure the number of files downloaded when a web page is viewed. A web page is typically made up of a number of individual files, such as HTML documents, images, JavaScript files. When a web page is viewed, each file is requested from the server, adding to the hit-count.

**Page views:** (or pages, impressions or URLs): a request to load a *single* HTML file (web page) of a web site, identified by the URL in a browser. During a single visit, several different pages may be accessed.

**Downloads:** measures the data downloaded from a resource in volume/bandwidth, often in GigabytesGB.

**Table.** The FAIR criteria mapped to the corresponding Core Data Resource indicators.

FAIR Principles	Core Data Resource Indicator(s)
*To be Findable:* F1 (Meta)data are assigned a globally unique and eternally persistent identifier. F2 Data are described with rich metadata. F3 (Meta)data are registered or indexed in a searchable resource. F4 Metadata specify the data identifier.	3a 1d, 3d 3f(i) 3a, 3d
*To be Accessible:* A1 (Meta)data are retrievable by their identifier using a standardized communications protocol. A1.1 The protocol is open, free, and universally implementable. A1.2 The protocol allows for an authentication and authorization procedure, where necessary. A2 Metadata are accessible, even when the data are no longer available.	3a, 3f(i,) 3f(ii) 3f(i), 4b 4b, 4c 4e
*To be Interoperable:* I1 (Meta)data use a formal, accessible, shared, and broadly applicable language for knowledge representation. I2 (Meta)data use vocabularies that follow FAIR principles. I3 (Meta)data include qualified references to other (meta)data.	2d, 3d, 3f(ii) 3d 2d, 3e
*To be Re-usable:* R1 (Meta)data have a plurality of accurate and relevant attributes. R1.1 (Meta)data are released with a clear and accessible data usage license. R1.2 (Meta)data are associated with their provenance. R1.3 (Meta)data meet domain-relevant community standards.	1d, 3d 4b 2d, 3d, 3e 3d

Box 2 presents a ‘Case Document’ template for describing a data resource using these indicators.

### Indicators to support expert judgment

Taking into account that ‘Not everything that can be counted counts, and not everything that counts can be counted’ (William Bruce Cameron ^5^), the indicators will be used to inform a peer-review process described below.

A carefully chosen set of qualitative and quantitative indicators, tailored to bioinformatics resources, will inform identification of the ELIXIR Core Data Resources. The indicators will support, but not supplant, expert judgment.

ELIXIR Core Data Resources should each have an international independent Scientific Advisory Board. Such boards are made up of distinguished academic and industry researchers and professionals who conduct scientific and/or technological review, ensuring quality and providing strategic advice to resource managers. Identification of ELIXIR Core Data Resources does not encroach on these governance structures. The establishment of Scientific Advisory Boards for Core Resources and Nodes is among the best practices that will be promoted by the Node Capacity Building and Communities of Practice.

Indicators can only be useful if they are underpinned by an open, transparent and coherent collection infrastructure, so clear methods of collection and processing are needed.

### Seed list of ELIXIR Core Data Resources

Using the definition of ELIXIR Core Data Resources above, we identified a ‘seed list’ of candidate core resources ( Table 2) to inform Core Data Resource indicators.

**Table 2.** Examples of European data resources that are considered as core for the life-science community.

Resource name	Institutions	Type
UniProt	EMBL-EBI (European Molecular Biology Laboratory – European Bioinformatics Institute); SIB Swiss Institute of Bioinformatics; Protein Information Resource (PIR) - Georgetown University Medical Centre	Knowledgebase of proteins
European Nucleotide Archive (ENA)	EMBL-EBI, in the framework of the International Nucleotide Sequence Database Collaboration (INSDC)	Comprehensive archive of nucleotide sequences, annotations and associated data
PRIDE (Proteomics identifications database)	EMBL-EBI	Archive of mass spectrometry based proteomics data
Europe PubMed Central (Europe PMC)	EMBL-EBI	Archive for full-text biomedical and life- sciences journal articles
InterPro	Consortium of databases based at EMBL-EBI, EMBL Heidelberg; SIB Swiss Institute of Bioinformatics; WTSI; University of Manchester; PRABI; J. Craig Venter Institute, Rockville; PIR; University of Bristol; University College London; University of Southern California	Knowledgebase of protein families, each represented by multiple sequence alignments and hidden Markov models (HMMs)
Protein Data Bank in Europe (PDBe)	EMBL-EBI, in collaboration with the Worldwide Protein Data Bank (wwPDB) and EMDataBank partners	Protein structure knowledgebase
Human Protein Atlas	AlbaNova and SciLifeLab, KTH - Royal Institute of Technology, Stockholm, Sweden, the Rudbeck Laboratory, Uppsala University, Uppsala, Sweden and Lab Surgpath, Mumbai, India	Knowledgebase with high-resolution images showing the spatial distribution of proteins in normal human tissues and cancer types, as well as human cell lines

### Identifying ELIXIR Core Data Resources

Identification of ELIXIR Core Data Resources involves a careful evaluation of the multiple facets of the data resources. This paper describes the overall approach for the selection of Core Data Resources, which will evolve over the coming months as the principles described in this paper are put into practice for the first time.

Indicators used are described in Box 1. The relevant ELIXIR Node submits the completed ‘Case Document’ ( Box 2) to the ELIXIR Hub.

Only data resources that are part of an ELIXIR Node Application and/or Service Delivery Plan (in the case of EMBL-EBI, the ‘Work Programme’) can be candidate ELIXIR Core Data Resources.

Initial evaluation of the ELIXIR Core Data Resources takes place annually.

The ELIXIR Hub checks the Case Document for completeness and verifies whether the proposed Resource is included in the Node’s Service Delivery Plan (or Work Programme). The ELIXIR Hub has an advisory role in selecting ELIXIR Core Data Resources. The Hub has no decision-making power and does not evaluate the proposals.

The ELIXIR Director informs the Heads of Nodes Committee of the candidate ELIXIR Core Data Resources. The Heads of Nodes Committee can request additional information about a candidate Resource from the relevant Head of Node.

The Heads of Nodes Committee convenes annually in person to review submitted Case Documents and determine the list of ELIXIR Core Data Resources. The initial selection is expected to grow with time.

The ELIXIR Scientific Advisory Board will review the process in March 2017. In this initial test process and evaluation, up to 20 ELIXIR Core Data Resources will be selected.

Based on the experience from this first selection round, the Heads of Nodes Committee may recommend a refinement of the indicators and the overall process for future. Should the process prove to be sufficiently robust, the selected candidate resources will become the first set of ELIXIR Core Data Resources. The ELIXIR Scientific Advisory Board also reviews the portfolio of ELIXIR Core Data Resources and provides ongoing advice on the process for their identification.

As each ELIXIR Core Data Resource already has a governance structure that includes an independent, international Board, this individual review is not duplicated by the ELIXIR Advisory Board. The outcome is presented to the ELIXIR (governance) Board for information and to ensure that the process has been correctly applied.

Through the work of the Nodes, Advisory Board and the ELIXIR Hub, standardized data on indicators can also be collected and monitored.

In collaboration with the Nodes, monitoring data will be automatically collected at the ELIXIR Hub on an ongoing basis and will be regularly transmitted to the Heads of Nodes. Nodes undertake to provide the necessary data to the specification defined.

### Reviewing the ELIXIR Core Data Resources

ELIXIR Core Data Resources may be requested to report regularly on certain indicators, and to provide updates on any major changes.

The Heads of Nodes meeting will review all ELIXIR Core Data Resources every two to three years. However, a minimum of three Heads of Nodes may request an extraordinary evaluation of an individual resource, in particular, on the basis of the monitoring data. If the review raises issues concerning an ELIXIR Core Data Resource, the Heads of Nodes Committee is responsible for determining what action should be taken.

## Discussion

### Supporting the sustainability of data resources and how defining identifying them will contribute to science policy

ELIXIR Core Data Resources form the centre of ELIXIR’s sustainability strategy. The collected key indicators for these bioinformatics resources, and more specifically the impact and translational stories, will be used to make a case to funders. This information will in turn help them to translate the impact that Core Data Resources make.

Impact evaluation attempts to provide a definitive answer to the question of whether the resource is meeting its objective of fulfilling a specific need of the scientific community. The translational stories relate to the role of the resource in accelerating science and are thus a very important indicator.

In addition, the ELIXIR Core Data Resources could contribute to impact and econometric analysis of life-science data within ELIXIR, as well as events focused on communicating the value of sustainable infrastructure for open data to the European Commission and other stakeholders.

### Capacity building

Core Data Resources will act as flagships of excellence. The use of defined indicators, in particular those around user policies and procedures, will be useful as benchmarks of quality and will support capacity building within the ELIXIR Community.

For example, the ELIXIR Core Data Resources, especially the knowledge bases, can function as ’concept authorities’ within and beyond ELIXIR, having a clear role in standardising what the community understands by a given biological concept.

Certain additional indicators could be used outside of ELIXIR (e.g. uptime) to consolidate confidence across a wide range of stakeholders. This would require full transparency on how indicators are produced, so as to avoid misunderstanding or misuse.

### Life-cycle management

Key indicators will inform life-cycle management, identifying trends and supporting decision-making around a given resource. This is important not only for the resource teams, but also for identifying Emerging Services that may evolve into ELIXIR Services. As new resources are listed in the ELIXIR Node Service Delivery Plans, indicators and capacity building around the Core Data Resources will support Emerging Services as they mature.

### Underpinning actions to support long-term sustainability and integration with ELIXIR Services

ELIXIR Core Data Resources will be prioritised for technical actions and for training. ELIXIR Core Data Resources become the primary resources for ELIXIR Cloud, storage and data distribution efforts within the ELIXIR Nodes network. These actions will be important for supporting the evolution of Emerging Services associated with Core Data Resources.

ELIXIR will strive to add value to all ELIXIR resources, including ELIXIR Services, by supporting interactions of the Core Data Resources with one another and with ELIXIR Services and Emerging Services for the benefit of the larger user community. Examples of this are use-case driven enhancement of the interoperability of the ELIXIR Core Data Resources with one another and with other ELIXIR Services, supporting helpdesks to scale national operations, and implementation studies to explore links to national infrastructures and data services.

## Conclusion

ELIXIR Core Data Resources form the centre of ELIXIR’s sustainability strategy and science policy actions. The collected key indicators reflect the diversity of these bioinformatics resources, and will be used to make a case to funders. This information in turn will help them to translate the impact that Core Data Resources make.

Key indicators for Core Data Resources, in particular those around user policies and procedures, will be useful as flagships of excellence and best practice to support capacity building within the ELIXIR Community. The process may be extended to incorporate best practices on interoperability, on concept naming, identifier resolution, identifier mappings and data identity provision and protection.

The key indicators will inform life-cycle management, identifying trends and supporting decision-making around a given resource. This is important not only for the teams managing the resources, but also for the identification of Emerging Services that may evolve into Core Data Resources. As new resources are listed on the ELIXIR Node Service Delivery Plans, the indicators and capacity building around the Core Data Resources will support the growth of Emerging Services as they mature.

As ELIXIR continues to mature, the framework for life-cycle management will be put into practice, supporting the Emerging Services, and strengthening the ELIXIR infrastructure by creating a stairway to excellence.

The use of both quantitative and qualitative indicators reflects the need to understand the context in which resources operate, providing a clear and rational basis for efforts to strengthen resources and improve capacity building. Establishing the portfolio of ELIXIR Core Data Resources and ELIXIR Services is a key priority for ELIXIR and publicly marks the transition towards a cohesive infrastructure.

Box 2. **Case Document Template**

A ‘Case Document’ describes a (candidate) Core Data Resource and is based on the indicators introduced in Box 1.

**Case Document: [Resource Name] v1.0**

**Document owner: [Insert Name] [email address]**

**1. Scientific focus and quality**

a. **Archival vs knowledge base:** is the resource
  • archival (taking submissions)
  • knowledge base (added-value)
b. **Scope statement**: describe the scientific coverage and comprehensiveness of the resource. For example, all species or a subset of species, families, outputs from a particular experimental method? How is the resource positioned with respect to other similar data resources?
c. **International dimension:** does the resource have a global footprint? (e.g. demonstrated through an international consortium delivering the resource, geographical diversity in the source of the submissions, global literature curated, international diversity of delivery partners and/or funders)
d. **Staff effort:**

**Table:** 

Number of FTE	[Year 1]	[Year 2]	[Year 3]
***Curators*** • **support for submission adherence to metadata requirements** • **support for extraction of information from the scientific literature** ***Bioinformaticians*** ***Technical staff***

**2. Community**

a. **Overall usage - quantitative:** what is the usage of the resource for the past 2–3 years?
      Please indicate the method used to derive these indicators.
**Access via a web browser (using web analytics, example: Google Analytics**

**Table:** 

Average monthly web traffic	[Year 1]	[Year 2]	[Year 3]
**Visits (sessions)**			
**Unique visitors (users)**			
**Page views**
**Access via a web browser (using log analytics)**

**Table:** 

Average monthly web traffic	[Year 1]	[Year 2]	[Year 3]
**Unique visitors (users)**			
**Hits**			
**Sessions and pages (if possible)**
**Data downloads (FTP, APIs, etc.)**

**Table:** 

Average monthly downloads	[Year 1]	[Year 2]	[Year 3]
**Hits/Requests**			
**Unique IP addresses/Hosts**			
**Data transfer (GB)**
b. **Potential usage:** what is the estimated size of the global potential user community?
c. **Usage in research as measured through citation in the literature:**
  Please indicate the method used to derive these indicators.

**Table:** 

Annual totals:	[Year 1]	[Year 2]	[Year 3]
**Resource name mentioned in Europe PMC (citation of resource name)**		
**Accession numbers mentioned in Europe PMC (citation of data of the resource)**
**Key publications describing the resource list (e.g. publications in NAR Database issue) and the number of citations (in Europe PMC):**
d. **Dependency of other resources:**do other resources depend on the resource described here to provide that service (i.e. what is the reach through)? Please list.

**3. Quality of service**

a. **Identifier use:** does the resource provide persistent and unique identifiers?
b. **Data throughput:** number of entries, depositions (records or bytes ingested per year), records processed, genomes assembled, etc. annually for past 2–3 years.

**Table:** 

	[Year 1]	[Year 2]	[Year 3]
**Total number of entries/depositions**			
**Size in GB**			
**Size (other)**
c. **Technical performance**:
  i. **Uptime:** percentage availability per month for a sample of key web pages (or similar) over the past 12 months (e.g. search results, homepage, data record pages).
  ii. **Response times of key web pages**.
d. **Use of standards:** which community-recognised standards are used for metadata and data (e.g. MIAME, JATS, INSDC features, ontologies)? Provide a link to documentation.
e. **Links to documentation of provenance:** does the resource link to the scientific literature for provenance of facts or biological context?
f. **Data availability – access services and formats:**
  i. **Data sharing services:** list services through which data is shared (e.g. website, APIs, FTP, TripleStore)
  ii. **Data sharing formats:** list formats data is available in (e.g. text, FASTA, XML, Dublin Core, tsv, JSON)
g. **Customer service**:
  i. **Helpdesk:** does the resource operate a helpdesk?
  ii. **User feedback:** does the resource seek and incorporate user input into service design decisions?
  iii. **Training:** does the resource undertake training activities?

**4. Legal and funding infrastructure, governance**

a. **Scientific Advisory Board**: does the resource have an international, independent Scientific Advisory?
b. **Open Science**: does the resource have a legal framework that supports Open Science? e.g. open licenses or public statement of open terms of use.
c. **Privacy policy**: does the resource have a publically available privacy policy in which security around personal data and cookies are described?
d. **Ethics** **policy:** does the resource have an ethics policy that complies with all relevant international standards and best practices?
e. **Sustainable support and funding**: demonstrate the past and future funding commitments and/or other commitments that support the resource by the host institution and/or other entities.

**5. Impact and translational stories**

a. **Counterfactual:** what would the impact on the scientific community be if the resource had not existed or was to disappear and not be replaced? Is the resource globally unique? What would the impact on other dependent resources be?
b. **Accelerating science:** how does the resource accelerate science? For example, does the resource set standards; promote reuse of data or software; promote research efficiencies; extend technical products in other areas?
c. **Translational data:** are there ‘translational’ figures that are familiar to the audience that will help them grasp the core nature of the resource?
