# Supplementary material for: Identifying ELIXIR Core Data Resources
Source: F1000Res. 2017 Mar 9;5:ELIXIR-2422. Originally published 2016 Sep 30. [Version 2] doi: 10.12688/f1000research.9656.2 (PMC5070591; doi:10.12688/f1000research.9656.2)
Supplement: Peer review history

# Peer review: Referee response for version 1

**DOI:** 10.5256/f1000research.10405.r16722

**Published:** 2016-10-18

**Authors:** Maryann E. Martone

**Affiliations:** 1 Department of Neurosciences, Center for Research in Biological Systems, University of California, San Diego, La Jolla, CA, USA; 2 Hypothes.is, San Francisco, CA, USA; 3 Founder, SciCrunch.com, San Diego, CA, USA

I understand that this article is included in the Elixir report collection and it does, in fact, read like a report.  If one is very familiar with Elixir, it probably makes sense, but if one isn't, then it is a bit confusing to read.

 But it contains valuable information that I think would be generally useful to everyone trying to develop methods for evaluating data resources.  In fact, an RFI put out by the US National Institutes of Health on repository metrics just closed today.  So if the authors are willing, I think that providing some modifications would make the report more readable to a general audience. 

1. It would be nice if the first paragraph introduced Elixir a bit more and explained its structure.  This could be done through either a diagram or a reference.  But the Hub idea is critical to the governance of Core Data Resources proposed and it would be nice to make the structure clear.
2. The tense of the article is a bit unclear.  Are there already approved Core Data Resources that have been evaluated by the criteria outlined?  At times, it seems that way and other times, it seems like the process has not yet been implemented.  In Table 2, some examples that are considered "core" are given.  But in the text, it says that the resources in table 2 were identified as a "seed list" to inform Core Data Resources.  So it implies that they haven't yet gone through the process.  I think stating up front where you are in the process would make it less confusing.
3. Methods section:  The term "indicator" is first used in second paragraph of the first section of methods section, but is not defined until the next section.  It should be defined earlier.
4. "Legacy stage" is used in the 3rd paragraph.  Legacy has a meaning in data-already existing-and so I think some definition is required here.  It is, in fact, defined in Table 1, so a reference to the Table would be sufficient.  
5. The indicators are listed in the main text, again in Box 1 and again in Fig 2.  Seems like a bit of overkill.  Also, the purpose of the indicators is defined in multiple places and it is a bit repetitive, e.g., the explanation given under the section "Detailed description of the indicators and related methodology" really isn't necessary.
6. FAIR is introduced in Box 1, but not in the text at all.  It seems like it should be mentioned in the introduction to indicators in the methods as it is used as a set of criteria throughout Box 1. 
7. Is there a timeline as to when the first core resources will be approved (if they haven't been already-see pt 2)

I have read this submission. I believe that I have an appropriate level of expertise to confirm that it is of an acceptable scientific standard.

# Peer review: Referee response for version 1

**DOI:** 10.5256/f1000research.10405.r16871

**Published:** 2016-10-10

**Authors:** Helen Berman

**Affiliations:** 1 Department of Chemistry & Chemical Biology, Center for Integrative Proteomics Research, Rutgers, The State University of New Jersey , Piscataway, NJ, USA

This is a very well written article describing how ELIXIR Core Data Resources are identified and evaluated.  The figures, tables, and illustrative boxes have been carefully designed and add to the clearly written text. This paper should be required reading for every panel and funding agency tasked with evaluating these resources.

I have read this submission. I believe that I have an appropriate level of expertise to confirm that it is of an acceptable scientific standard.
